# Furry, Feral, Foe: Temporalizing Heath and Invasion on an English Chalk Stream

**DOI:** 10.1093/jhmas/jrae043

**Published:** 2024-12-09

**Authors:** Maddy Pearson

**Affiliations:** London School of Hygiene & Tropical Medicine, London, UK

**Keywords:** Invasive species, feral ecologies, chalk streams, health, environment, temporalities

## Abstract

This article explores framings of life, death, health, and invasion on an English chalk stream. It focuses on the ways in which these notions have been put to work in recent history, in relation to each other, and in relation to particular species and spaces. By 2019, narratives of a chalk stream in South-East England as a dead river expanded beyond retort to intermittent waterlessness. The river’s death came to be framed as part of a wider ecology of chalk stream (ill)health, influenced by twenty-first century biodiversity conservation narratives and hauntological effects, which rendered deathly chalk stream futures present and requiring of human-action now. These narratives and effects conditioned a powerful sense of which non-human life belonged and counted, and which non-human life did not. Absent flagship chalk stream species, water voles, and efforts to resurrect them, were made synonymous with restoring the river itself to life and health. Contrarily, the ongoing presence of “invasive” American mink served as a continued reminder of the river’s demise and death as a chalk stream. The resurrection of chalk streams to health relied on their being dispatched. Once considered to belong as extracted “lively capital” dominating the fur industry and later tolerated as feral escapees in the wild of the UK, American mink had been resituated and their history progressively obscured. Humans became manager-come-saviors of chalk streams, whose lost health was agreed and rendered visible through the ghostly image of the water vole that must be saved from the invasive foe, American mink.

The River Beane is nestled in the countryside of Hertfordshire, South-East England. It winds through the hills of Sandon, traversing picturesque villages until reaching the county town of Hertford where it acts as a tributary to the River Lea. The presence of water has been recognized as giving meaning to rivers.^1^ Of particular note both within and beyond Western culture has been the associations among river water, flow, and life.^2^ The River Beane is no exception, and for most interlocutors I met during my fieldwork in 2019, its status as a living entity depended on the presence of flowing water. This river was, however, further layered with understandings of life as a chalk stream. There are only 200 or so chalk streams globally, 85% of which can be found in the United Kingdom.^3^ Chalk streams are fed by underground chalk aquifers and their waters are purified from percolating between levels of chalk.^4^ The minerally cleansed water of chalk streams acts as the optimum habitat for the water vole *Arvicola terrestris*, brown trout *Salmo trutto*, European otter *Lutra lutra,* and invertebrates such as mayfly *Ephemeroptera* and winged olive *Serratella ignita*.^5^ Thus life for a chalk stream extends beyond water’s presence to include particular non-human species.^6^ This framing of chalk stream life has been inflected by conservation science, its recent historical focus on biodiversity loss, and a “‘construction’ of the biodiversity crisis as a new environmental problem.”^7^ This orientation was cemented in the politico-legal sphere through Section 40 and sub-section 41 of the Natural Environment and Rural Communities Act of 2006 (NERC) that recognized chalk streams as habitats of principal importance for wildlife, and chalk stream species including water voles as protected species.^8^

That life for the River Beane is considered in relation to water, chalk, and non-human species indicates that living waters are multiple.^9^ However when I began investigating this river in 2019, I was told by river restoration groups, wildlife charities, and individual environmental activists that the River Beane was dead or dying. The River Beane did have increasing stretches of waterlessness, but in places still flowed. These groups and individuals told me that the River Beane had lost many of the native non-human species associated with chalk streams such as water voles, but did have native invertebrates as well as further species, American mink *Mustela vison*, that I was instructed were non-native invasives. How then was the River Beane made sense of at this time, as a river that escaped, expanded, and threatened imaginaries of flow? What constituted a dead river? For whom? And what role did species which were native and absent, or invasive and present, play in constructions of chalk stream life and death? Finally, following the provocation of Celia Deane-Drummond, how was this story of a diminished river system being told, and by which humans? How did these stories foster actions and what were the ethics at play?^10^

Exploring these questions, I argue that a narrative of death on the River Beane was used by some interlocutors to demarcate the absence of life it was felt should belong, in particular the water vole, and life that was present that it was felt did not belong, American mink. I trace practices I observed on the River Beane that enacted this issue as a kind of “catastrophic thinking” (to borrow David Sepkoski’s term) of an alien species, invading, disrupting natural balance, and threatening the health of chalk streams as an imagined pristine entity of nature.^11^ This leads me to consider what was rendered opaque when nature was framed in separation from culture and thus de-temporalized. What of American mink’s history – its journey to the UK to supply the fur industry? Where did this more-than-human assemblage that had spanned a century sit in relation to recent historical debates of invasiveness and river death? Finally, noting that on the River Beane water voles were entirely absent by 2019, I ask what happened when American mink completely overran the water vole habitat, resulting in a local extinction whereby mink invasion was framed, in a sense, as complete? How were notions of saviorism, resurrection, and practices of restoration put to work in order to reverse said invasion and bring a river, and the non-humans made synonymous with its health (water voles), back to life? What was the role here of spectrality – how did absent water voles haunt the present, and in turn, garner political support and action to save *our* chalk streams?^12^

Drawing together ethnography, archival research, and literature from anthropology, blue humanities, and ecology, in this article I demonstrate that by 2019, the river’s death came to be framed as part of a wider ecology of chalk stream (ill)health, influenced by twenty-first-century biodiversity conservation narratives and hauntological effects, which rendered deathly futures present and conditioned a powerful sense of which non-human life belonged and counted and which non-human life did not.^13^ This revealed how the early-twentieth-century history of American mink as extracted “lively capital” for the fur industry, and their positioning as feral when they escaped, or were released, and first established themselves in the wild of the UK, had been progressively obscured.^14^

Biodiversity is a complex, historically contingent, and flexible concept, and its meaning is slippery and nebulous, having served diverse scientific, political, and economic purposes since its coinage in 1986.^15^ In the case of the River Beane, conservationists, politicians, and local community groups have argued the necessity of its protection on account of its unique geology and ecology, its charismatic native species, and the importance of both for English national heritage. Framing their arguments in terms of an ongoing catastrophe – the “death” of this river and the elimination of its charismatic fauna by invasive species – these individuals became managers and saviors of unique English biodiversity (defined as richness and variety of *native* species). In turn, the declining health of chalk streams as ecosystems was agreed upon and rendered visible through the ghostly image of the water vole, in need of salvation from what over time had been repositioned as that which did not belong at all, the invasive foe, American mink.

## Flows of Life and Death

**Figure 1:**
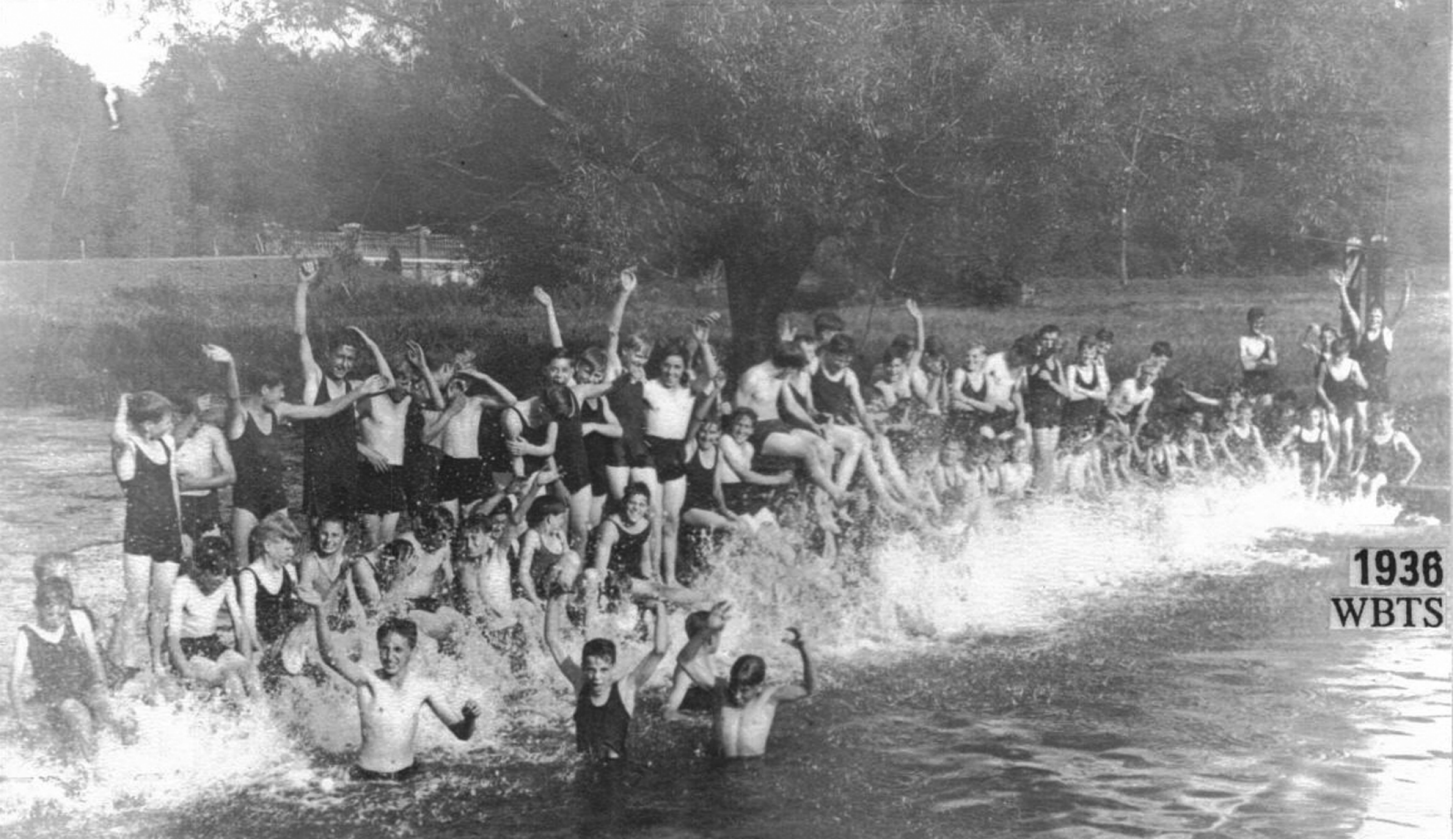
Boys learn swim in the River Beane in 1936. Reproduced with permission of Golding Old Boys Association.

Rivers are permeated with liveliness in the widest sense; they are constituent parts of the “hydro-modernities” through which life and power come to be entwined.^16^ Rivers’ waters have been encapsulated in public health histories, with river bathing recognized in the fifth century BC as bestowing vitality, and being encouraged in England between 1700-1920s for health, understood holistically as physical, mental, and spiritual wellbeing.^17^ These associations are evidenced through the River Beane’s industrial and public health history, where it served as the main source of drinking water in Hertfordshire, powered the first ever paper mill in England, referenced in the Domesday book of 1086, and was popular for river bathing in the nineteenth and twentieth centuries (see Figure 1).^18^

Given river waters’ dominant association with life, a smaller body of work in the humanities has attended to the spectre of death such spaces carry. Flow can symbolize life entering but also death leaving the city, and a single river has been found to hold dual meaning as holy and pure while also polluted and polluting.^19^ Moving beyond the symbolic to consider more-than-human assemblages, anthropologists have recognized the deep-seated politics of the polluted river “in us,” and how this threatens health, existence, and place-based identities built through centuries of multispecies river-based relations.^20^ Historians have asked what is in store for *Rivers of the Anthropocene,* thinking about life and death for rivers in relation to increasingly uncertain environmental futures.^21^

## Uncertain Parameters of Chalk Stream Health

The equating of water flow with river life punctuated conservation efforts until the emergence of biodiversity conservation as a key concern in the late 1980s. This trajectory was mirrored in my fieldwork. On a cold November morning, I first met with members from the River Beane Restoration Association (RBRA). This group of individuals, mostly local residents, formed in 1991 having perceived worryingly low river water levels. At this time, restoration “just meant getting water back in.”^22^ The group monitored groundwater levels each month, sending a tape measure deep underground, measuring the gap between the ground beneath their feet and the water beneath the ground. They submitted these numbers to the Environment Agency (EA) and used them to lobby for water abstraction reduction by the local water company that supplied drinking water from the aquifer. After nearly fifteen years, the water company proposed to reduce abstraction at a pumpstation on the River Beane’s aquifer by 90%. This was accepted by the Water Services Regulations Authority (OFWAT), and in 2014 abstraction was reduced. To the group’s disappointment and confusion, river water levels did not bounce back. In fact, longer stretches of the river dried up.

This situation put the river restoration group in a bind. They remained deeply concerned about the river’s health, so worried that they described the river as dying. This “death” was doubly sad because, as Allan McNab, a member of the RBRA put it, chalk streams are a “unique and irreplaceable part of our national landscape and heritage,” and are “rarer than giant pandas: they are our rainforests, our most endangered national habitats.”^23^ However, how this impending death, understood as a lack of flowing water, was linked to the river’s source, the chalk aquifer, was increasingly uncertain. The group surmised that the 10% abstraction still taking place was the problem. Hydrologists at the local water company refuted this, disagreeing that a dry river was a dead one. They explained the River Beane as having a hyporheic zone, a damp layer beneath the channel where life could survive without the flow of continuous water.^24^ Thus for the water company, flow was not the only parameter of chalk stream health and life. They saw life in a more sedimentary fashion, as present in the “muddyscapes” of the waterless riverbed.^25^ These debates over what counted as life on the river continued and made it harder for the group to rally support on the basis of life made akin to flow.

Despite the uncertainty, the RBRA were not alone in trying to raise the plight of the River Beane and trying to make its (ill)health and possible death count. They had the support of some of their local Members of Parliament (MPs). The MP for Broxbourne felt that chalk streams were not glamourous enough to garner widespread political attention or capture public imagination. The public cared more about saving the rainforest – these supposedly untouched, sublime, and wild landscapes were spaces in which biodiversity conservation was important – not English rivers with long industrial histories. Of course, they did not realize that chalk streams were their home grown equivalent.^26^ For the chairman of the RBRA, Dave Stimpson, the River Beane’s death was premised on a series of disconnections extending beyond public disconnection and disinterest to include abdicated responsibility by water companies, governmental bodies, and regulatory agencies.

In the face of disconnection and uncertainty, and with divergent parameters of life and death being espoused by different actors in relation to the River Beane, the RBRA, local wildlife charities, and individual environmental activists recognized the need for something more certain around which to orient their restoration activities. They needed to find a parameter or signifier of life and death that could unite and foster agreement across different river-involved parties and garner not just support, but also action to save the River Beane.

## Re-orienting Efforts – Saving Ratty

The RBRA expanded their concerns over the River Beane’s death and directed attention towards the absence and presence of certain non-human species. Roni Edmonds-Brown, an aquatic ecologist at the University of Hertfordshire, for example, who was interviewed in the RBRA’s documentary *On the Banks of the Bean* (2014), connected the loss of water flow and the absence of native species. Commenting on proposals from a water company to reduce water abstraction from the River Beane by 90%, she argued that despite this measure increasing water supply, “as to recovery there are problems with this, it’s not going to be an instant. Just because you’ve got water there doesn’t mean you’re going to have everything occurring the following year. It will take at least five, maybe as much as eight years to get to a diversity and a list of species that you’d expect to see in a chalk river.”^27^ This connection between the river and its native species was politico-temporal; it spoke to an emerging environmental political movement around restoration, conservation, and nativeness, reflecting growing concerns about species invasiveness as a driver of biodiversity loss.^28^

The group began to orient their restoration activities around the water vole. The water vole functioned as a charismatic species, that is, one around which public sympathy for conservation action could be inculcated due to characteristics that are cute, cuddly, or easily anthropomorphized.^29^ The water vole had been entrenched in British culture as an affable and loveable character through the protagonist Ratty from the classic children’s storybook, *The Wind In the Willows*.^30^ The restoration of water voles was more feasible than that of other native chalk stream species. Otters would have required the restoration of an entire underlying ecosystem, and brown trout would have struggled to reproduce in the River Beane given the historical emplacement of weirs and sluice gates which hindered upstream swimming to spawn and could not, as legally protected listed structures, be removed in many cases.

The water vole’s successful candidacy extended beyond charisma and feasibility. The water vole had a nemesis around which restoration could be narrated and framed as well as actively practiced. The UK does not have a native species of mink, which is what usually serves to denote invasiveness in broader public imaginaries. Instead, American mink that had established themselves in the wild of the UK since their escape or deliberate release from fur farms were framed by the 2010s as an increasingly invasive threat to the health of chalk stream ecosystems by way of their predation of native water voles.^31^ Depicted in this way, American mink offered an opportunity for all involved human actors, river restoration groups and beyond, to act as potential saviors of the river’s health. They could begin the process of restoring the River Beane by restoring a native species (water voles), and actively culling a health-threatening invasive one (American mink). For example, Peter Creasey, an RBRA member stated on interview that, “the water voles have not been so apparent lately, I’m quite worried about them.” When questioned as to whether mink were the source of the trouble, he stated that “there are mink around,” and that in one area they had to “dispose of some mink that appeared.”^32^ Another member, Robert Thornton, claimed that they were “particularly concerned about…the control of invasive species” such as “mink and signal crayfish, Himalayan balsam, and giant hogweed.”^33^

The way mink and voles were described contributed to this positioning. The World Wildlife Fund (WWF) report on England’s chalk streams labelled water voles “vulnerable” and “timid.”^34^ American mink on the other hand were described across varied literature sources as an invasive alien species, “aggressive predators” that “will overkill prey.”^35^ The proliferation of this species was framed as a catastrophic driver of biodiversity loss within England. Recourse was made not only to its “unusual reproductive physiology,” its propensity for “superfecundation and superfoetation” serving to further contribute a sense of alienness, but also to its insatiable appetite for mammals.^36^ This combination of a somewhat alien ability to reproduce quickly in large numbers and to eat in insatiable amounts (phenomena definitive of vermin species in the early modern British empire), situated American mink as “the main agents of destruction,” providing “an incriminating mirror image to the decline of water voles.”^37^ The absence of water voles came to be framed less in relation to anthropogenic change and was increasingly framed in relation to American mink.^38^

## American Mink – From Fur, Through Feral, to Invasive Foe

American mink were exported to the UK in 1929 for purposes of mink farming, which supplied the growing fur fashion industry.^39^ Mink was popular not only for its warmth-giving properties but throughout the mid-twentieth century as a sign of wealth, luxury, and femininity, allowing mink to “arouse the interest of many people… even dignified ladies.”^40^ The exploitation of fur-bearing mammals incentivized some early exploration in North America and by the early 1900s White settlers and trappers had become the major supplier of animal pelts.^41^ The wild supply of American mink was fast depleted as the industry grew, thus mink ranches were established in North America to breed mammals in captivity.^42^ Ranch-bred mink were exported to the UK as European mink, *Mustela lutreola*, were smaller, lighter colored, and less densely furred, making them less preferable for fashion than the large American mink that sported darker fur of a finer texture.^43^ Captivity produced accidental mutant minks with unusual colour variations such as the Silverblu and Platinuum, which became popular among consumers and became “the aberrant colour variety most frequently noted in feral populations in the UK.”^44^

Mink fur farming peaked in the UK in the 1950s with 400 known farms recorded.^45^ At this point, the world population of captive mink stood between ten and eleven million.^46^ Desire for fur contracted sharply across the UK in the latter decades of the twentieth century and by 1981 only sixty-seven farms remained in England, continuing to decline to twenty-seven operational mink farms in 1993.^47^ The environmental movement and animal rights campaigning were instrumental in this decline: having gained momentum, they strongly influenced public opinion in the 1980s and 1990s.^48^ Anti-fur organization Lynx teamed up with Greenpeace in 1984 to commission the “40 Dumb Animals Campaign.” A woman is depicted dragging a fur coat along the floor. The slogan alongside the trail of crimson-coloured blood she leaves reads: “It takes up to 40 dumb animals to make a fur coat and only one to wear it.”^49^ A follow-up Lynx public survey found 71% agreed it was wrong to kill animals for fur, and 70% agreed that trapping, rearing, and farming animals for fur should be banned.^50^

On 30 July 1998, a House of Commons debate reflected on a public consultation in England and Wales and found “overwhelming support to ban fur farming.”^51^ The government spent two subsequent years developing legislation with the Fur Farming Prohibition Act coming into effect in 2000.^52^ This banned fur farming, restricted fur imports for England and Wales and was extended to Scotland and Northern Ireland in 2002.^53^ The demise of fur farming did not spell the end for American mink. Escapes and deliberate releases by animal rights activists from fur farms during their years of operation led to American mink establishing themselves in the wild, first sighted in England in the 1930s and confirmed to be breeding there in 1956.^54^ Thus, American mink remained present, outliving their human-defined purpose and as it would soon become clear, outstaying their spatially specific welcome.

As early as 1962, the Ministry of Agriculture and Fisheries (MAF) and the Ministry of Agriculture, Fisheries and Food (MAFF) published guidance on “wild” or “feral” American mink, encouraging their “control” or “destruction.”^55^ These policies demonstrated political awareness of American mink’s presence in the wild of the UK shortly after their breeding there was first confirmed. It also demonstrated that the presence of American mink in the UK was never, in their entire history living beyond the confines of fur farms, fully accepted. Yet paying attention to the language used to describe mink and their presence outside fur farms over the last sixty years is interesting. Shifts in this discourse demonstrated the temporal and dynamic nature of what was classified as belonging and also rendered opaque that invasiveness, non-nativeness, and foreignness, as means to determine which species life counted and represented the (ill)health of chalk stream environments, are confined to recent history.

From the 1960s to the late 1980s American mink that escaped or were released from fur farms were denoted most often as “feral,” “escaped,” and sometimes “wild.”^56^ Publications in ecology from this period used the term “feral” and questioned rather than asserted the impact of American mink on British wildlife.^57^ The Oxford dictionary defines feral in simple terms, as an animal “in a wild state, especially after escape from captivity or domestication.” And yet, as Barua demonstrates of parakeets in London “as a concept with plural connotations, the feral brims with tensions.”^58^ Xenophobic, nationalist rhetoric positions the parakeet as an invasive “foreigner,” while at the same time affective practices of feeding in public parks animate the parakeet as a creature to share space and dwell with.^59^

American mink, as feral, encapsulated this plurality.^60^ How then since the 1990s, and particularly with the turn of the century, did framings of American mink shift and these creatures come to belong even less? How by the early 2000s was the plural potential of the feral constrained, and the feral cemented as foe? How by the 2010s had the presence of American mink been equated with other mammalian species’ and chalk stream death?

The term biodiversity was first coined in 1986 (as “BioDiversity”) at a conference held in Washington, DC. Used by numerous natural scientists, it is commonly attributed to Walter Rosen who used it as a contraction of biological diversity, recognizing in the early 1990s that the term could “help the cause of conservation with the general public.”^61^ Biodiversity as a way of understanding and framing ecosystems, species interactions, and habitat deterioration quickly became a cornerstone of conservation policy and practice, gaining momentum throughout the 1990s not just as a scientific concept but as a political slogan.^62^ Yet despite its widespread currency, the term biodiversity itself has been slippery, with scientists failing to “agree on a basic definition for biodiversity.” Its flexibility has enabled governments and advocacy groups to use it to push multiple aims.^63^ As a way of both knowing and in turn acting upon the environment, biodiversity reached dominance in the UK around 2006, being written into environmental policy through the NERC Act.^64^ However, this act fails or avoids to define the term biodiversity itself, deferring to the UN Convention on Biological Diversity of 1992, which defines “*Biological diversity*” as “the variability among living organisms from all sources including, *inter alia,* terrestrial, marine and other aquatic ecosystems and the ecological complexes of which they are part; this includes diversity within species, between species and of ecosystems.”^65^

Despite this lack of clear definition, the NERC act nevertheless frames biodiversity as a kind of resource that can be “gain[ed]” or lost and quantified. It is something that must be conserved but also can be “enhanced,” presumably through the encouragement of greater numbers of local species.^66^ It also facilitates the conservation of charismatic, flagship species, mandating that the “Secretary of State must,” in consultation with Natural England, “publish a list of the living organisms and types of habitat which in the Secretary of State’s opinion are of principal importance for the purpose of conserving [or enhancing] biodiversity,” and may also require that these reports include “specified quantitative data relating to biodiversity in any area of land in England.”^67^

With growing concern about preserving, promoting, and quantifying diversity of indigenous species in England, so too were increasing amounts of money and research allocated to exploring the impact of non-native species on priority habitats.^68^ Denigrated chalk stream environments were growing in research interest and American mink were surfacing more frequently as a threat to the biodiversity of such spaces, due to their predatory status.^69^ American mink were included under section 51 of the NERC Act, which ordered the management and reporting of species that are “not ordinarily resident in and are not regular visitors to Great Britain in a wild state.”^70^ Thus within less than half a century, American mink had been repositioned three times, from that which belonged as a luxury product of the fur trade, to that which belonged less as a feral, semi-wild escapee of captivity, to that which did not belong at all, as a fearsome predator and embodiment of threats to chalk stream biodiversity and habitats writ large.

## Reversing a Complete Invasion? The Role of Ghostly Water Voles

On the River Beane, water voles were not just framed as being threatened by the presence of American mink, in turn symbolizing a threat to the life of the River Beane as a chalk stream. By 2019, water voles were no longer present on the River Beane at all. How then were water voles still put to work and made present even in their absence? How did this keep the hope of healthy chalk stream ecologies for the future alive? How was an invasion that was in a localized sense completed, invoked as one that could be reversed?

The presence of water voles in biodiversity discourse, visual media campaigns, and talks from wildlife charities, and their being offered as the reason behind stringent monitoring and culling of America mink, kept these rodents present as haunting figures. That conservation efforts are haunted by the ghosts of species and environmental landscapes has been recognized by scholars across the social sciences, particularly in the face of anthropogenic change and increasingly uncertain futures.^71^ Discussing the hauntological effect of ghost orangutans, Liana Chua explains “the capacity of edge imaginaries to galvanize: to compel their audience to take responsibility for an ape whose imminent extinction they make visually, and sometimes viscerally, thinkable.”^72^ A related but divergent conservation effort emerged on the River Beane where water voles were not on the edge so to speak, but were entirely gone: the mink had allegedly completely driven away the voles. If river conservation activists had framed the River Beane’s life as entirely synonymous with water vole presence or absence, then it was no longer dying but dead. Instead, though, this hauntological effect was put to work and it was implied that something could be done through human intervention to restore the River Beane to life. Through human mastery and management of nature, realized as the monitoring and culling of invasive American mink and the re-placing of water voles along the River Beane, humans could save the River Beane.

The ghostly water vole rallied an agreed sense between different parties of what would constitute life again for the River Beane as a chalk stream ecosystem, and an agreed sense that this strategy for reversing mink invasion would work. This contrasted with conservation biology literature, which has recognized that eradicating small invasive carnivores is difficult if not impossible to achieve in full.^73^ Further, efforts to cull American mink to levels considered manageable for the successful reintroduction of water voles have been poorly evaluated, and the few studies that have attempted this reintroduced water voles that were subsequently killed by American mink, an ethically concerning situation.^74^ Yet actors I met appeared unaware or unperturbed about this; they had found a sense of certainty around which to orient their concerns over the River Beane – native species’ decline – and would not be derailed by further potential uncertainties. They were going to cull American mink, resurrect water voles, and save the River Beane.

## Saviorism, Restoration, and Resurrection

Social scientists have noted that hope (traces of which can be contained in hauntological effects) functions in an ontological sense, not just as a way of understanding reality or imagining something better, but as a method or practice through which more positive futures might be produced in the face of “blasted landscapes.”^75^ Hope as a method was part of a four-step trajectory on the River Beane in 2019, pursued by the RBRA and conservation biologists, and supported by Commons policy and the UK’s largest canal charity, Canal & Rivers Trust. Firstly, they established American mink’s presence by monitoring stretches of the riverbank for tracks or droppings. Secondly, trapping mink highlighted them through monitoring and thus tangibly materialized them. Thirdly, the trajectory included dispatching of mink by shooting them. Fourth and finally, the goal was to reintroduce water voles sourced from rivers where they still resided. Water vole resurrection was part of this hopeful practice – it was a form of saviorism. How though could putting one creature to death be made akin to saving, situated not only as morally accepted, but in fact as a kind of environmental moral duty?

Saviorism as part of human-environmental relations on the River Beane, and in relation to water voles on chalk streams more broadly, should be understood within a wider history. Through the Renaissance a “constructed discontinuity between humans and nonhumans” emerged in Western Europe, equating the nonhuman with nature and the human with culture.^76^ This premised culture (humans) as higher status bearers of agency, juxtaposed with a non-agentic nature. This discontinuity has permeated ways of knowing and doing more-than-human relations since the fourteenth century and continued to inflect them in the twenty-first century.^77^ This resulted in a situation whereby the human (specifically the White male) remained as the only “plausible actor,” who from the privileged position of culture could intervene and manage “nature” as an entity from which he stood entirely separate.^78^ Social scientists have highlighted this as fiction, noting the cyborg nature through which affective more-than-human relations (animal and otherwise) emerge and create ways of being in the world.^79^ Furthermore, the popularizing of the Anthropocene narrative has worked to demonstrate the inextricable link among humans, nonhumans, and the environment.^80^ However, the “anthropos” has remained at the center in both narrative and practice. Other creatures and planetary entities occupy a space nearer and more relational than surmised during the Renaissance, but are still not ascribed agency in the same way and thus remain as entities that humans can, and most importantly should, intervene on.^81^

This human-centered rhetoric was enacted in the House of Commons’ 2019 debate, *Degraded Chalk Stream Environments*.^82^ MPs stated that management of “fragile ecosystems that are chalk streams” needed to be “perfect,” and reinforced chalk streams as “extraordinary features of *our natural world,*” something “*we manage.*”^83^ As well as indications of chalk streams as something belonging to humans, part of “*our* national heritage,” further language emerged at this time, cementing this sense of human ownership, management, and a sense of moral duty and obligation.^84^ The Canal & Rivers Trust described the “urgency of this situation,” while conservation biologists Anthony R. Martin and Vince J. Lea firmly stated “the only long-term solution to the ‘mink problem’ in Britain is to remove them entirely,” describing “the ecological benefits of mink eradication” as “profound.”^85^ As noted earlier, the lack of certainty around river flow as death led river-concerned groups such as the RBRA to question the orientation of their efforts. However, with American mink control there appeared to be no such uncertainty. The RBRA, local water company, wildlife charities, EA, all agreed and were supported by the legal provisions in the NERC Act that destroying American mink was the right way to both imagine and control what belonged and signified a threat to life both on and of a chalk stream.

Saviorism also worked through the media, and drew more individuals to the cause, advising that “any landowner or gamekeeper licensed to carry firearms is eligible to join the cull at designated trial areas,” killing mink with “a ‘clean’ shot to the head.”^86^ Wildlife charities such as the Sussex Wildlife Trust used less aggressive language to stress the importance of mink being “dispatched” after trapping.^87^ The Canal & Rivers Trust recognized this “humane mink control as an essential tool in water vole conservation, within the National Species Action Plan.”^88^ That the Canal & Rivers Trust took a strong stance on American mink culling demonstrates how deeply embedded the presence or absence of particular non-human species as indicator of chalk stream health had become.

There were two twists that hampered efforts to ready and reintroduce water voles on the River Beane at this time. Halfway along the river’s course, a listed weir structure became so clogged with debris in 2018 that minimal river water could pass through.^89^ Following a period of heavy rain, the river burst its banks above the weir and water supply downstream was almost entirely cut off. After months of monitoring, reporting, and ensuring American mink on the river were culled to a low enough density ready for water vole reintroduction, the uncertainty posed by low or no river flow due to the weir calamity put a halt to this plan. The local water company spent 2019 and 2020 building a bypass to the weir, allowing the river to meander around this structure. No sooner had this bypass been completed and restoration efforts put back on track, than the coronavirus (SARS-CoV-2) emerged as a serious threat to public health in England and a national lockdown was initiated by the British Government. All river monitoring activities ground to a halt and American mink spent the next year effectively un-mapping themselves. While finding spatial freedom from culling efforts in England at this time, American mink found themselves further embroiled in questions of health elsewhere in Europe. Having been identified as a reservoir for the coronavirus, captive American mink in Denmark and the Netherlands were subject to culls.^90^ This further cemented the mink’s identity as a foe by extending perceptions of their threat beyond the health of environmental landscapes and native mammals to include the health of humans.^91^ Outside of captivity, mink culling was also advocated “for the benefit of conservation and human health.”^92^

These events, the weir that burst the river’s banks and the coronavirus that derailed all river-based activities, did not deter belief from the RBRA and local wildlife charities that water voles (and river) could be resurrected. However, there was an irony to the two events: both resulted from the inextricable link among humans, nonhumans, and the landscapes and practices through which they interact and come to be affected by one another. These landscapes and pandemics were peppered with histories of industry and extraction and could not be separated from efforts to cull American mink and restore water voles. They were infused in this process, rendering futile any imaginary of nature and culture as separate. They undermined, although apparently not to the awareness of those involved, the narrative upon which this saviorism had been built, and forced questions of the ethical politics of this haunting – of whose ghosts, and which ghosts count.

## Temporalizing Invasiveness – Is It Time to be Haunted by Our Own Ghosts?

Saving water voles from American mink had been folded into a wider effort to save chalk streams. These efforts were spatio-temporally specific, as MP Charles Walker suggested when he stated, “saving the world starts right here, right now, doing our bit locally with our chalk streams — think locally, act globally.”^93^ The saviorism associated with water voles had been powerfully conditioned by concerns for protecting native species, ecology, and biota, which was of recent historical origin. This framing of chalk stream life and health had entrenched a sense of species that belong, native water voles, and rendered species such as American mink a deeper and more threatening embodiment of that which was invasive and did not belong. Throughout this paper I have worked to demonstrate the dynamic temporal nature of this interplay between species’ absence and presence, chalk stream life and death, and human practices of management, that worked to reverse a localized extinction, framed as the result of an invasion, to cull certain life-forms and restore others.

Temporalizing invasives such as American mink is not to undermine the importance of biodiversity conservation. Nor is it to refute that American mink have preyed on water voles and have no doubt put pressure on this species. It is however to recognize that invasiveness as a threat to native biodiversity is the lens through which American mink have become known in recent history, and by virtue of this, have come to both belong even less in spaces such as chalk stream environments and have also been considered not only to not belong, but also to threaten the health of these ecosystems to the point that their presence is equated with water vole and chalk stream death. This dominant way of knowing American mink and of materializing their presence as invasive through an infrastructural assemblage of legal doctrine, ecological science research and writing, media portrayals, citizen science practices of monitoring, the use of traps to capture these aliens and of shotguns to dispatch them is temporally dynamic, specific, and serves to shadow the extractive history of the fur trade that brought American mink to the UK in the first place. The fact that American mink exploitation and extraction was also part of a process of White settlers in North America, who went from trading with native Americans to enclosing land for ranches and exporting creatures around the world, is just another part and parcel of this extractive, exploitative history.^94^

The demise of the fur trade in the UK was in many ways a process of facing up to ghosts. The public were encouraged to think about the morality of their actions and were encouraged to consider the killing of multiple animals in the name of fashion as ethically unacceptable. And yet, what is so interesting is that the culling of American mink in the name of conservation continues to kill mink. This time, it is within a temporal, environmental-political moment of future uncertainty and biodiversity concern which allows the American mink to be portrayed as the morally bad party and those who put this entire species to death in the name of the health of chalk stream species and ecologies as morally good. Biodiversity conservation, as a practice of preserving or recreating an imagined pristine nature, a time when water voles were present on the River Beane, is positioned as something that can be saved by culture, yet all the while the true history of an inextricably linked nature and culture, of industries that have fused multispecies assemblage together across time and space, is obscured.^95^ Thinking locally to save chalk streams as a global action carries within it the irony that the saga of water voles and American mink that plays out locally is itself derives from global actions – from an international trade in “lively capital” and fur that has faded into the background of recent historical conservation efforts.^96^

The director of Animal Aid, Andrew Tyler, referred to this process as “scapegoating the aliens” and argued for attention to the “absurdity to aspire to environmental and/or genetic purity with regard to local fauna and flora” given that “humans’ reshaping of the environment is ‘endless.’”^97^ Tyler drew attention not only to physical changes wrought on environmental landscapes within the UK (the weirs that traverse the River Beane would be just one example), but as I have done through the case of American mink, to the international trade, transportation, and translocation of species that cannot be separated from such changes. Writing two decades earlier Nigel Dunstone, lecturer in evolutionary biology and conservation biology, had already tried to “explode the mink myth.”^98^ This “false belief that the mink is a destroyer of all living creatures, causing disaster in occupied habitats” diverted attention from the real factors affecting wildlife such as pollution and land use change.^99^ The fact that uncertainty as to the long-term impacts of efforts to cull American mink on the restoration of water voles remained, appeared to have been discounted on the River Beane. This was dissolved into small enough traces within a sea of agreement and thus did not destabilize overwhelming confidence across groups that biodiversity (and American mink culling) was the way to both understand and solve the problem of water vole absence and chalk stream death.^100^

## Conclusion: Indigenous Biodiversity and Hauntology

Introducing the collection *Arts of Living on a Damage Planet: Ghosts of the Anthropocene*, Elaine Gan, Nils Bubandt, Anna Lowenhaupt Tsing, and Heather Anne Swanson propose that ghosts and their haunting presence prevent us from forgetting the multiple pasts of a landscape even as it continues to morph.^101^ While water voles haunted the River Beane in 2019, it appeared that their spectral presence was part of selectively remembering particular, or creating imagined, rather than multiple pasts.^102^ The ghostly water vole on the River Beane had been put to work as part and parcel of an epistemologically privileged nature, that rendered some pasts (imagined pristine ones) more visible than others and shrouded some histories from view almost entirely. Conservation efforts on the River Beane remember a history it is hard to say has ever existed, one of pristine nature, from which humans appear curiously absent. In doing so these efforts also do something quite peculiar. They position invasion and invasive species as something both natural and alien. The story of present American mink and absent water voles by 2019 had become framed as an issue of nature, of two species battling against one another for life, with that life being subsumed within debates of another life hanging in the balance – that of the chalk stream, the River Beane, and the ecological health of chalk stream landscapes more widely.

While conservation efforts are haunted by the spectrality of ghost species such as the water vole, ghosts that entangle conservation in ways that are not only ecological but increasingly moral and political, the idea that ghost species can act as “productive conceptual tools” warrants critical engagement.^103^ While these ghosts did incite action, and could in such a sense encourage more-than-human connections, they allowed conservation efforts to be haunted by the good – haunted in ways that propel them to action, increasingly aggregated with saviorism. This selective remembering or conjuring of imagined histories allowed conservation efforts on the River Beane to escape being haunted by the bad, by the extractive industry of the trans-Atlantic fur trade and of American mink whose being forced to belong has over time been reframed as an invasive threat to health that must be destroyed.^104^ Thus, ghosts do not just allow particular actors concerned with river degradation to remember multiple histories, they also allow them to reframe and forge them, to continue, where it suits moral purpose, to hold nature and culture separate – a surprisingly impressive feat in an era preoccupied with anthropogenic change and its negative effects.

While I do not wish to undervalue the desperate need to protect indigenous biodiversity in the face of these uncertain futures or the heartfelt effort of those undertaking conservation work on the River Beane who care for and worry about its future, it has been my intention to demonstrate the histories of extraction that in present day conservation efforts of invasion and health are rendered less visible. If there is to be responsibility, saviorism, and pats on the back for resurrecting species and chalk stream spaces to health, then I posit there must also be awareness of the extractive industries that forced certain invasive species to exist out of place and to show remorse for deaths that could have been avoided. Histories of extraction must be rendered visible and held to account, to allow us to recognize that the framing of life and death of chalk streams in relation to species is related to the enviro-temporal politics of conservation discourse in recent history. It is based on one way of knowing American mink, as that which brings death to other mammalian species, symbolizes death of wider ecologies such as chalk streams, and as that which can only be met with death, framed as a form of human-enforced, humane saviorism.

The philosopher and integrative biologist Sahotra Sarkar writes, “epistemological considerations return to haunt us when we take seriously the obligation to act and begin devising strategies to protect the environment. Moreover, now they often become intertwined with ethical and aesthetic ones.”^105^ In the case of the River Beane, Sarkar’s words provoke those of us who identify as English, or identify with the English landscape, to be haunted by our own ghosts. Our own changing ways of knowing American mink, most commonly today as invasive, foreign aliens, posing a deathly threat to chalk stream species and habitats, creatures that it is our duty to destroy – is just one way in which American mink have been known. On this dynamic, temporally sliding scale of not-belonging, within a history of extraction and exploitation, mink were within the last century considered free in North America, farmed as that which was luxuriously furry in the UK, feral upon first escape, and by 2019 foe. Whether mink survive long enough to experience further inscriptions, whether they can be lived with, rather than against, and whether such inscriptions might function as part of a wider shift in biopolitical thinking remains to be seen.

## Footnotes

1 Veronica Strang, *The Meaning of Water* (Oxford: Berg, 2003).

2 Ibid.; Georgina Drew, “Why Wouldn’t We Cry? Love and Loss along a River in Decline,” *Emotion and Ecology* 6 (2013): 25–32.

3 The Wildlife Trust, “Chalk Rivers,” n.d., https://www.wildlifetrusts.org/habitats/freshwater/chalkrivers#:~:text=Only%20200%20chalk%20rivers%20are,in%20southern%20and%20eastern%20England. There is disagreement over exactly how many chalk streams exist globally. The most commonly cited number is 200, but estimates range from 200-230.

4 A.D. Berrie, “The Chalk-Stream Environment,” *Hydrobiologia* 248 (1992): 3–9. Chalk-fed rivers are chalk streams and are sometimes referred to in literature and the media as chalk rivers.

5 Ibid.

6 Peter Fox, “Protect Our Precious Chalk Streams,” *Environment Agency Blog*, 2 October 2019, https://environmentagency.blog.gov.uk/2019/10/02/protecting-our-precious-chalk-streams/.

7 Esa Väliverronen, “Biodiversity and the Power of Metaphor in Environmental Discourse,” *Science & Technology Studies* 11 (1998): 19–34.

8 “NERC Act,” § 41 (2006), https://www.legislation.gov.uk/ukpga/2006/16/section/41.

9 Franz Krause and Veronica Strang, “Introduction to Special Issue: ‘Living Water,’” *Worldviews* 17 (2013): 95–102.

10 Celia Deane-Drummond, “Rivers at the End of the End of Nature: Ethical Trajectories of the Anthropocene Grand Narrative,” in *Rivers of the Anthropocene* (Berkeley: University of California Press, 2020), 55–62.

11 For this term as a way of thinking about biodiversity loss and extinction, see David Sepkoski, *Catastrophic Thinking: Extinction and the Value of Diversity from Darwin to the Anthropocene* (Chicago: University of Chicago Press, 2020).

12 Author’s ethnographic observations with the Chalk Aquifer Alliance who stressed chalk streams through the possessive term “our” (2019).

13 Marianne E Lien, “‘King of Fish’ or ‘Feral Peril’: Tasmanian Atlantic Salmon and the Politics of Belonging,” *Environment and Planning D: Society and Space* 23 (2005): 659–671.

14 Maan Barua, “Feral Ecologies: The Making of Postcolonial Nature in London,” *Journal of the Royal Anthropological Institute* 28 (2022): 896–919. See Barau’s discussion of Donna Haraway’s expression “lively capital” in relation to “invasive” parakeets in London that formed part of the trade in exotic animals, 900.

15 Sepkoski, *Catastrophic Thinking*, chapter 6.

16 Erik Swyngedouw, *Liquid Power: Contested Hydro-Modernities in Twentieth-Century Spain* (Cambridge, MA: MIT Press, 2015); John Lansing, *Priests and Programmers* (Princeton: Princeton University Press, 1991).

17 Wilbert M. Gesler, “Therapeutic Landscapes: Medical Issues in Light of the New Cultural Geography,” *Social Science & Medicine* 34 (1992): 735–746; Elizabeth Archibald, “Bathing, Beauty and Christianity in the Middle Ages,” *Insights, Durham University Institute of Advanced Study* 5 (2012): 2–13; Christopher Love, “The Reflecting Pool of Society: Aquatic Sport, Leisure and Recreation in England, C 1800-1918,” (PhD diss., University of York, 2003), 23-37.

18 “Fascimile of the Portion of Domesday Book Relating to Hertfordshire,” 1863, DE/X12/47, Hertfordshire Archives and Local Studies.

19 Jonathon Parry, *Death in Banaras* (Cambridge: Cambridge University Press, 1994); Terje Oestigaard, *Death and Life-Giving Waters: Cremation, Caste, and Cosmogony in Karmic Traditions* (Oxford: Archaeopress, 2005).

20 Catherine Fennell, “Are We All Flint?” *LIMN*, Public Infrastructures/Infrastructural Publics, no. 7 (2016), https://limn.it/articles/are-we-all-flint/; Elizabeth Hoover, *The River Is in Us* (Minneapolis: University of Minnesota Press, 2017).

21 Jason M. Kelly, “Anthropocenes: A Fractured Picture,” in *Rivers of the Anthropocene*, ed. Jason M. Kelly, Philip Scarpino, Helen Berry, James Syvitski, Michel Meybeck (Berkeley: University of California Press, 2018), 1–18.

22 Author’s ethnographic fieldnotes, 2019.

23 “On the Banks of the Beane” video (2014), *RBRA YouTube Channel,* uploaded 14 June 2020, https://www.youtube.com/watch?v=ebxGxaqeuR4.

24 Author’s interview notes with water company hydrologists, 2019-2020.

25 Flore Lafaye de Micheaux, Jenia Mukherjee, and Christian A Kull, “When Hydrosociality Encounters Sediments: Transformed Lives and Livelihoods in the Lower Basin of the Ganges River,” *Environment and Planning E: Nature and Space* 1 (2018): 641–663.

26 Author’s ethnographic observations and interview notes. Houses of Parliament, Westminster, January 2020.

27 “On the Banks of the Beane.”

28 Väliverronen, “Biodiversity and the Power of Metaphor.”

29 Jamie Lorimer, *Wildlife in the Anthropocene: Conservation after Nature* (Minneapolis: University of Minnesota Press, 2015), 44-50.

30 “The Animals of The Wind in the Willows,” *BBC*, 10 January 2005, seg. Inside Out, https://www.bbc.co.uk/insideout/southwest/series7/wind_in_willows.shtml.

31 Tom Porteus, Mike Short, Suzanne Richardson, Jonathan Reynolds, “Empirical Development of Strategy for the Control of Invasive American Mink by Trapping,” *European Journal of Wildlife Research* 58 (2012): 403–413.

32 “On the Banks of the Beane.”

33 Ibid.

34 WWF, “The State of England’s Chalk Streams,” 2014, http://assets.wwf.org.uk/downloads/wwf_chalkstreamreport_final_lr.pdf. 3, 26.

35 Laura Bonesi and Santiago Palazon, “The American Mink in Europe: Status, Impacts, and Control,” *Biological Conservation* 134 (2007): 470–483; “American Mink,” *Norfolk Wildlife Trust*, n.d., https://www.norfolkwildlifetrust.org.uk/wildlife-in-norfolk/species-explorer/mammals/american-mink, para. 3.

36 David Whyte Macdonald and Lauren A. Harrington, “The American Mink: The Triumph and Tragedy of Adaptation out of Context,” *New Zealand Journal of Zoology* 30 (2003): 421–441.

37 Ibid., 423-424; Lucinda Cole, *Imperfect Creatures: Vermin, Literature, and the Sciences of Life, 1600-1740* (Ann Arbor: University of Michigan Press, 2016), 4.

38 Nigel Dunstone, *The Mink* (London: T. & A.D. Poyser, 1993), 193.

39 C. Douglas Deane and Fergus O’Gorman, “The Spread of Feral Mink in Ireland,” *Irish Naturalists’ Journal* 16 (1969): 198–202.

40 Marcin Brzenziński, “The Mink,” *Acta Theriologica* 39 (1994): 109–111.

41 Dunstone, *Mink*, 164.

42 Ibid.

43 Deane and O’Gorman, “Feral Mink in Ireland”; Dunstone, *Mink*.

44 Dunstone, *Mink*, 167.

45 “Mink in Britain,” Game & Wildlife Conservation Trust, n.d., https://www.gwct.org.uk/wildlife/research/mammals/american-mink/mink-in-britain/, para. 4.

46 Deane and O’Gorman, “Feral Mink in Ireland,” 198.

47 Dunstone, *Mink*, 167.

48 Carly Halliday and Steven P. McCulloch, “Beliefs and Attitudes of British Residents about the Welfare of Fur-Farmed Species and the Import and Sale of Fur Products in the UK,” *Animals: An Open Access Journal from MDPI* 12 (2022): 1-18.

49 Janice Li, “Lynx Dumb Animals Campaign,” *V&A* Blog, 17 January 2019, https://www.vam.ac.uk/blog/projects/dumb-animals-lynxs-campaign-against-the-fur-industry.

50 Dunstone, *Mink*, 186.

51 “Fur Farming,” House of Commons, 30 July 1998, https://api.parliament.uk/historic-hansard/commons/1998/jul/30/fur-farming, 317.

52 “Fur Farming Prohibition Act,” § 33 (2000), https://www.legislation.gov.uk/ukpga/2000/33/contents.

53 “Fur Farming (Prohibition) (Scotland) Act,” § 10 (2002), https://www.legislation.gov.uk/asp/2002/10/contents; “Fur Farming (Prohibition) (Northern Ireland) Act,” § 3151 (2002), https://www.legislation.gov.uk/nisi/2002/3151/contents.

54 Kaarina Kauhala, “Introduced Carnivores in Europe with Special Reference to Central and Northern Europe,” *Wildlife Biology* 2 (1996): 197–204; Dunstone, *Mink*, 23.

55 “Destruction of Escaped Mink” (Ministry of Agriculture and Fisheries, 1962-1965), MAF 131/86, The National Archives, United Kingdom (TNA); “Control of Wild Mink: Policy,” Ministry of Agriculture and Fisheries, 1962-1965, MAF 131/230, The National Archives, United Kingdom (TNA).

56 Deane and O’Gorman, “Feral Mink in Ireland”; Paul Chanin, “Observations on Two Populations of Feral Mink in Devon, U.K.,” *Mammalia* 47 (1983): 463–476; “Control of Wild Mink: Policy.”

57 Chanin, “Feral Mink in Devon.”

58 Barua, “Feral Ecologies,” 898.

59 Ibid., 904, 911.

60 “Pest Control: Wild Mink on Your Land?” Ministry of Agriculture and Fisheries, n.d., MAF 217/8, The National Archives, United Kingdom (TNA).

61 Sahotra Sarkar, “Origin of the Term *Biodiversity*,” *BioScience* 71 (2021): 893.

62 Väliverronen, “Biodiversity and the Power of Metaphor.”

63 Sepkoski, *Catastrophic Thinking,* chapter 6. See also David Takacs, *The Idea of Biodiversity: Philosophies of Paradise* (Baltimore: Johns Hopkins University Press, 1996).

64 NERC Act. See Section 40.

65 United Nations, *Convention on Biological Diversity* (Montreal: Secretariat of the Convention on Biological Diversity, 2011), 4. Note despite its publication date this pamphlet contains “the text and annexes of the Convention on Biological Diversity, first adopted 22 May 1992” (see Front Matter).

66 “Natural Environment and Rural Communities Act 2006,” part 3, https://www.legislation.gov.uk/ukpga/2006/16/enacted, accessed 9 September 2024.

67 Ibid., part 3, section 41.

68 Ibid., part 3, section 51.

69 Bonesi and Palazon, “American Mink in Europe.”

70 NERC Act. See section 51, Wildlife and Countryside Act 1981, section F84-85, https://www.legislation.gov.uk/ukpga/1981/69/section/51.

71 Liana Chua, “Edgy Imaginaries: ‘Ghost’ Orangutans, Extinction, and Responsibility in a Plantation Landscape,” in *Anthropology and Responsibility*, ed. Melissa Demian, Mattia Fumanti, Christos Lynteris (London: Routledge, 2023), 13–36; Adriana Petryna, “Wildfires at the Edge of Science: Horizoning Work amid Runaway Change,” *Cultural Anthropology* 33 (2018): 570–595.

72 Chua, “Edgy Imaginaries,” 16.

73 Daniel Simberloff, “Eradication of Island Invasives: Practical Actions and Results Achieved,” *Trends in Ecology & Evolution* 16 (2001): 273–274.

74 Lauren A. Harrington, Andrew L. Harrington, Tom Moorhouse, Merryl Gelling, Laura Bonesi, David W. Macdonald, “American Mink Control on Inland Rivers in Southern England: An Experimental Test of a Model Strategy,” *Biological Conservation* 142 (2009): 839–849.

75 Hirokazu Miyazaki, *The Method of Hope: Anthropology, Philosophy, and Fijian Knowledge* (Stanford: Stanford University Press, 2004); S. Eben Kirksey, Nicholas Shapiro, and Maria Brodine, “Hope in Blasted Landscapes,” *Social Science Information* 52 (2013): 228–256.

76 Joanna Latimer and Mara Miele, “Naturecultures? Science, Affect and the Non-Human,” *Theory, Culture & Society* 30 (2013): 5–31.

77 Ibid.

78 Gunther Teubner, “Rights of Non-Humans? Electronic Agents and Animals as New Actors in Politics and Law,” *Journal of Law and Society* 33 (2006): 497–521; Tim Ingold, *The Perception of the Environment: Essays on Livelihood, Dwelling and Skill* (London: Routledge, 2011).

79 Donna J. Haraway, *When Species Meet* (Minneapolis: University of Minnesota Press, 2008).

80 Hannah Gibson and Sita Venkateswar, “Anthropological Engagement with the Anthropocene: A Critical Review,” *Environment and Society* 6 (2015): 5–27.

81 Astrida Neimanis, Cecilia Asberg, and Johan Hedren, “Four Problems, Four Directions for Environmental Humanities: Toward Critical Posthumanities for the Anthropocene,” *Ethics & the Environment* 20 (2015): 67–97.

82 “Degraded Chalk Stream Environments,” House of Commons, 22 July 2019, https://hansard.parliament.uk/commons/2019-07-22/debates/180ABF92-245D-4855-A2BF-A13212E3162C/DegradedChalkStreamEnvironments, emphasis by the authors.

83 Ibid., column 1178, emphasis by the authors.

84 Author’s ethnographic fieldnotes, 2019.

85 Anthony R. Martin and Vince J. Lea, “A Mink-Free GB: Perspectives on Eradicating American Mink Neovison Vison from Great Britain and Its Islands,” *Mammal Review* 50 (2020): 170–179.

86 Mark Townsend, “Mink Face Cull to Save Ratty from Wipe-Out,” *Guardian Online*, 2 March 2003, https://www.theguardian.com/uk/2003/mar/02/politics.greenpolitics, para. 7.

87 “Mink Control Best Practice for Conservation,” Sussex Wildlife Trust, March 2016, https://dnu7gk7p9afoo.cloudfront.net/mink-control-best-practice-1.pdf, 5.

88 “American Mink,” Canal & Rivers Trust, n.d., https://canalrivertrust.org.uk/enjoy-the-waterways/canal-and-river-wildlife/canal-and-river-invasive-species/american-mink, para. 6.

89 Listed structures are objects classified as having historical interest. They are closely controlled by Historic England and cannot be removed without express permission.

90 Florence Fenollar, Oleg Mediannikov, Max Maurin, Christian Devaux, Philippe Colson, Anthony Levasseur, Pierre-Edouard Fournier, Didier Raoult, “Mink, SARS-CoV-2, and the Human-Animal Interface,” *Frontiers in Microbiology* 12 (2021): 1–12, 2.

91 Carsten Schade Larsen and Søren Riis Paludan, “Corona’s New Coat: SARS-CoV-2 in Danish Minks and Implications for Travel Medicine,” *Travel Medicine and Infectious Disease* 38 (2020): 1–2.

92 Lauren A Harrington, María Díez-León, Asunción Gómez, Andrew Harrington, David W. Macdonald, Tiit Maran, Madis Põdra, Sugoto Roy, “Wild American Mink (*Neovison vison*) May Pose a COVID-19 Threat,” *Frontiers in Ecology and the Environment* 19 (2021): 266–267, 267.

93 “Degraded Chalk Stream Environments,” 1168.

94 Dunstone, *Mink*, 164.

95 Bruce Braun, “Environmental Issues: Global Natures in the Space of Assemblage,” *Progress in Human Geography* 30 (2006): 644–654.

96 Barua, “Feral Ecologies,” 902.

97 Andrew Tyler, “Scapegoating the Aliens,” Animal Aid, n.d., https://www.animalaid.org.uk/scapegoating-the-aliens/.

98 Dunstone, *Mink*, 188.

99 For similar arguments in the case of the sea lamprey, see Vincent Bijman’s paper, this volume. Brzenziński, “Mink,” 110.

100 Teubner, “Rights of Non-Humans?”

101 Elaine Gan, Nils Bubandt, Anna Lowenhaupt Tsing, Heather Anne Swanson, “Introduction: Haunted Landscapes of the Anthropocene,” in *Arts of Living on a Damaged Planet: Ghosts and Monsters of the Anthropocene*, ed. Anna Lowenhaupt Tsing, Nils Bubandt, Elaine Gan, Heather Anne Swanson (Minneapolis: University of Minnesota Press, 2017).

102 Chua, “Edgy Imaginaries,” 6.

103 Shane McCorristine and William M Adams, “Ghost Species: Spectral Geographies of Biodiversity Conservation,” *Cultural Geographies* 27 (2020): 101–115; Ben Garlick and Kate Symons, “Geographies of Extinction: Exploring the Spatiotemporal Relations of Species Death,” *Environmental Humanities* 12 (2020): 296–320.

104 Tyler, “Scapegoating the Aliens.”

105 Sahotra Sarkar, *Biodiversity and Environmental Philosophy: An Introduction* (Cambridge: Cambridge University Press, 2005), 6.

